# ASCL1 regulates proliferation of NG2‐glia in the embryonic and adult spinal cord

**DOI:** 10.1002/glia.23344

**Published:** 2018-04-23

**Authors:** Demetra P. Kelenis, Emma Hart, Morgan Edwards‐Fligner, Jane E. Johnson, Tou Yia Vue

**Affiliations:** ^1^ Department of Neuroscience University of Texas Southwestern Medical Center Dallas Texas; ^2^ Department of Pharmacology University of Texas Southwestern Medical Center Dallas Texas; ^3^ Department of Neurosciences University of New Mexico Albuquerque New Mexico

**Keywords:** ASCL1, NG2‐glia, OPCs, oligodendrogenesis, proliferation, spinal cord

## Abstract

NG2‐glia are highly proliferative oligodendrocyte precursor cells (OPCs) that are widely distributed throughout the central nervous system (CNS). During development, NG2‐glia predominantly differentiate into oligodendrocytes (OLs) to myelinate axon fibers, but they can also remain as OPCs persisting into the mature CNS. Interestingly, NG2‐glia in the gray matter (GM) are intrinsically different from those in the white matter (WM) in terms of proliferation, differentiation, gene expression, and electrophysiological properties. Here we investigate the role of the transcriptional regulator, ASCL1, in controlling NG2‐glia distribution and development in the GM and WM. In the spinal cord, ASCL1 levels are higher in WM NG2‐glia than those in the GM. This differential level of ASCL1 in WM and GM NG2‐glia is maintained into adult stages. Long‐term clonal lineage analysis reveals that the progeny of single ASCL1+ oligodendrocyte progenitors (OLPs) and NG2‐glia are primarily restricted to the GM or WM, even though they undergo extensive proliferation to give rise to large clusters of OLs in the postnatal spinal cord. Conditional deletion of *Ascl1* specifically in NG2‐glia in the embryonic or adult spinal cord resulted in a significant reduction in the proliferation but not differentiation of these cells. These findings illustrate that ASCL1 is an intrinsic regulator of the proliferative property of NG2‐glia in the CNS.

## 1 INTRODUCTION

Oligodendrocyte precursor cells (OPCs) are one of the most proliferative cell types in the central nervous system (CNS) and are specifically marked by the expression of platelet‐derived‐growth‐factor‐receptor α (PDGFRα; Pringle, Mudhar, Collarini, & Richardson, 1992) and the chondroitin sulfate proteoglycan NG2; and hence are commonly referred to as NG2‐glia (Butt et al., 1999; Nishiyama, Chang, & Trapp, 1999; Nishiyama, Komitova, Suzuki, & Zhu, 2009); (Dimou & Gallo, 2015; Levine, 2015). During development, NG2‐glia are widely distributed throughout all areas of gray matter (GM – containing neuronal cell bodies) and white matter (WM – containing axon fiber tracts) and readily differentiate to give rise to oligodendrocytes (OLs), which are responsible for myelinating axon fibers. Remarkably, a large number of NG2‐glia remain “undifferentiated” as OPCs throughout life, and have been shown to directly interact with neurons (Ge et al., 2006; Lin & Bergles, 2004b; Lin & Bergles, 2004a). These properties imply that NG2‐glia serve more than just a reservoir of OPCs to replenish turnover OLs, but may be a functionally unique class of glia within the CNS (Nishiyama et al., 2009; Richardson, Young, Tripathi, & McKenzie, 2011).

A unique feature of NG2‐glia is their ability to rapidly respond to local signals elicited from the environment. For instance, NG2‐glia can be stimulated by neuronal activity, injury, cell death, or disease states to migrate, proliferate, and if necessary, differentiate to form new OLs (Binamé, Sakry, Dimou, Jolivel, & Trotter, 2013; Bu, Banki, Wu, & Nishiyama, 2004; Hamilton et al., 2016; Hughes, Kang, Fukaya, & Bergles, 2013; Kang, Fukaya, Yang, Rothstein, & Bergles, 2010; Levine, 2015; Wake, Lee, & Fields, 2011; Wake et al., 2015; Young et al., 2013). Indeed, local induction of NG2‐glia proliferation and differentiation by neuronal activity to re‐myelinate projection neurons has been shown to be a critical step for the learning of complex motor skills and behaviors in mice (Gibson et al., 2014; McKenzie et al., 2014; Xiao et al., 2016). Thus, NG2‐glia not only serve as a source to supply the necessary OLs to maintain the function and metabolic needs of neurons but also to stabilize the plasticity and dynamics of neuronal circuits.

Initially, NG2‐glia was presumed to be a homogenous cell population throughout the various regions of the CNS, mostly because of a shared expression profiles of OL‐lineage markers such as OLIG1/2, SOX10, and PDGFRα (Lu et al., 2002; Nishiyama et al., 2009; Stolt, Lommes, Friedrich, & Wegner, 2004; Zhou & Anderson, 2002). However, with the advent of transgenic mice, extensive characterization of NG2‐glia *in vivo* revealed that they are far more heterogeneous than previously appreciated. For instance, electrophysiological recordings of labeled NG2‐glia in the cortical GM demonstrate that they exhibit distinct membrane potentials and expression profiles of potassium (K+) and sodium (Na+) channels than their respective counterparts in the subcortical WM or corpus callosum (Chittajallu, Aguirre, & Gallo, 2004). Similarly, GM NG2‐glia in the brain and spinal cord, whether during neonatal development or at adult stages, are generally less proliferative, differentiate at a slower pace, and respond differently to platelet‐derived‐growth‐factor (PDGF) in comparison to WM NG2‐glia (Dimou, Simon, Kirchhoff, Takebayashi, & Gotz, 2008; Hill, Patel, Medved, Reiss, & Nishiyama, 2013; Kang et al., 2010; Kang et al., 2013; Psachoulia, Jamen, Young, & Richardson, 2009; Rivers et al., 2008; Zhu et al., 2011). Transplantation experiments suggest that GM and WM NG2‐glia are intrinsically unique (Vigano, Mobius, Gotz, & Dimou, 2013), which may be directly related to their function within these regions in the CNS. However, at present it is unclear how the intrinsic properties of NG2‐glia in the GM or WM are regulated, or whether NG2‐glia in the GM are derived from the same or a separate OLP lineage than those in the WM.

Previously, we and others reported that the bHLH transcription factor ASCL1, which is well known for its roles during neurogenesis, is broadly expressed in glial progenitor cells throughout the ventricular zone (VZ) at the onset of gliogenesis in the spinal cord and in the cortex (Nakatani et al., 2013; Parras et al., 2007; Sugimori et al., 2007; Sugimori et al., 2008; Vue, Kim, Parras, Guillemot, & Johnson, 2014). Notably, ASCL1 expression is maintained in NG2‐glia as they migrate out of the ventricular zone to populate the surrounding GM and WM, but is downregulated once NG2‐glia differentiate to become mature OLs (Nakatani et al., 2013; Vue et al., 2014). Accordingly, *Ascl1* mutant and conditional‐knock out mice exhibit a significant decrease in the number of NG2‐glia and OLs that are generated, particularly in the WM of the spinal cord (Battiste et al., 2007; Nakatani et al., 2013; Parras et al., 2007; Sugimori et al., 2007; Sugimori et al., 2008; Vue et al., 2014), suggesting that ASCL1 may play an important role in regulating the generation of NG2‐glia in the CNS. However, the precise function of ASCL1 specifically in NG2‐glia during embryonic development or in the adult CNS remains unclear.

In this study, we show that the level of ASCL1 is substantially higher in WM NG2‐glia than in GM NG2‐glia during development of the spinal cord. Furthermore, clonal analysis using *Ascl1^CreERT2^* knock‐in mice carrying the stochastic multicolor *R26R‐loxP‐stop‐loxP‐Confetti* reporters (*R26R^LSL‐Confetti^*; Schepers et al., 2012; Snippert et al., 2010) demonstrate that progeny from single ASCL1+ OLPs labeled at embryonic day 14.5 (E14.5) are spatially restricted to the GM or WM, even though they proliferate extensively to form large clusters of SOX10+;Confetti+ OLs in the adult spinal cord. This suggests that OLPs are restricted in fate to the GM or WM by the time they express ASCL1. Finally, conditional deletion of *Ascl1* specifically in labeled (tdTomato+ or Confetti+) NG2‐glia using *Ng2‐CreER^TM^* mice at E14.5 or adult postnatal day (P) 30 resulted in a significant decrease in the proliferation, but not differentiation, of NG2‐glia. Taken together, these findings illustrate that the level of ASCL1 plays an important role in ensuring the proper generation of the number of NG2‐glia in the GM and WM of the spinal cord.

## 2 MATERIALS AND METHODS

### 2.1 Mouse strains

Generation and genotyping of mouse strains were performed as previously described: *Ascl1^CreERT2^* [Ascl1^tm1.1(Cre/ERT2)Jejo^/J] (Kim, Ables, Dickel, Eisch, & Johnson, 2011); *Ascl1^F^* [*Ascl1^flox^*] (Andersen et al., 2014; Pacary et al., 2011); *Ng2‐CreER^TM^BAC* [B6.Cg‐Tg(Cspg4‐cre/Esr1*)BAkik/J, JAX Stock # 008538] (Zhu et al., 2011); *Olig1^Cre^* (Lu et al., 2002; Xin et al., 2005); and the Cre reporter lines *R26R^LSL‐tdTOM^* [Gt(ROSA)26Sortm^14(CAG‐tdTomato)Hze^/J] (Madisen et al., 2010) and *R26R^LSL‐^* ^Confetti^ [Gt(ROSA)26Sor^tm1(CAG‐Brainbow2.1)Cle^/J 013731) (Snippert et al., 2010). All animal procedures followed NIH guidelines and were approved by the UT Southwestern Institutional Animal Care and Use Committee.

### 2.2 Tamoxifen and BrdU administration

The appearance of a vaginal plug was considered E0.5 and the day of birth was noted as P0. Tamoxifen (T5648, Sigma‐Aldrich Co., St. Louis, MO 63103, United States) was dissolved in 10% ethanol/90% sunflower oil mixed and administered via intraperitoneal injection. For population analysis using *Ng2‐CreER^TM^* crossed with *R26R^LSL‐tdTOM^* mice, a single dose of 2.5 mg tamoxifen/40 g body weight was injected to pregnant females at E14.5, or double doses of 2.5 mg tamoxifen/40 g body weight per day were injected to adult mice for two consecutive days starting at P30. For clonal analysis using *R26R^LSL‐Confetti^* mice, a single dose of 2.5 mg tamoxifen/40 g body weight was injected into pregnant females when crossed with *Ascl1^CreERT2^* mice, and single dose of 0.625 mg tamoxifen/40 g body weight was injected into pregnant females at E14.5 or adult mice at P30 when crossed with *Ng2‐CreER^TM^* mice. Due to the effects of tamoxifen on birth complications, cesarean section was performed on the day of birth (E19 or P0) on pregnant females that were injected with tamoxifen at E14.5. Pups were carefully introduced and raised by a foster female until date of analysis.

BrdU (Roche 10280879001) was dissolved at 5 mg/ml in 0.007 M NaOH. *Ng2‐CreER^TM^*; *R26R^LSL‐tdTOM^* mice that received tamoxifen injection at P30 were administered intraperitoneally with 3 mg BrdU/25 g body weight per day for 10 consecutive days starting at 7–16 days post‐tamoxifen injection.

### 2.3 Tissue preparation and immunofluorescence

Mice were cardiac‐perfused with 4% paraformaldehyde in PBS. Spinal cords were dissected and immersed in 4% paraformaldehyde in PBS overnight, washed in PBS and submerged in 30% sucrose. Tissues were embedded in O.C.T. for cryosectioning at 30 μm collected onto slides. Slides were incubated with primary antibody in 1% goat or donkey serum/0.3% Triton X‐100/PBS at 4°C overnight, followed by incubation with secondary antibody conjugated with Alexa Fluor 488, 568 or 647 (Molecular Probes). For spinal cord sections subjected for BrdU staining, dsRed antibody was used along with Alexa Fluor 568 secondary to amplify the fluorescent of tdTOM. Sections were then fixed in 4% PFA/PBS for 5 min, treated with 2 M HCl/0.2% Triton‐X100 for 20 min to exposed BrdU epitope, then incubated with anti‐BrdU overnight, followed by Alexa Fluor secondary the following day to detect BrdU+;tdTOM+ cells. All slides were mounted with Vectashield mounting medium (#101098‐042) and imaged using a Zeiss LSM 510 META and Nikon ECLIPSE 80i Upright fluorescent microscopes. The following antibodies were used for this study:

**Table:** 

Primary Antibodies	Source & Catalogue Number	Dilution
Chicken Anti‐GFP	Chemicon, AB16901	1:500
Goat Anti‐SOX10	R&D Systems, AF2864	1:20
Guinea Pig Anti‐ASCL1	Kim et al., 2008, TX518	1:1,000–1:10,000
Mouse Anti‐CC1 (or APC)	Calbiochem, OP80	1:100
Mouse Anti‐GFAP	Sigma, G3893	1:500
Mouse Anti‐CNP	Sigma, C5922	1:300
Mouse Anti‐MAG	Sigma, SAB1402258	1:300
Mouse Anti‐MBP	Calbiochem, NE1019	1:300
Mouse Anti‐NEUN	Chemicon, MAB377	1:1,000
Rabbit Anti‐dsRed	Clonetech #632496	1:500
Rabbit Anti‐OLIG2	Millipore, AB9610	1:1,000
Rat Anti‐PDGFRα (APA5)	BD Pharmingen, 558774	1:100

### 2.4 Quantification and statistical analysis

To quantify the immunofluorescence of ASCL1 in OLIG2+;PDGFRα+ NG2‐glia, ImageJ was used to draw an outline around the ASCL1 signal of each cell in the GM and WM of high magnification images (TIFF) of P2 or P30 spinal cord sections as described (McCloy et al., 2014). The area, integrated density, and mean fluorescence of each cell were measured, along with several readings of surrounding mean background fluorescence in the GM or WM. The total corrected cellular fluorescence (TCCF) = integrated density – (area of selected cell × mean fluorescence of background readings) was then calculated. The TCCF of cells in the GM was then compared with TCCF of those in the WM, as well as across stages using a Student's *t* test (GraphPad Prism 7.0c).

Quantification of the number of tdTOM‐labeled NG2‐glia lineage cells and area of the GM and WM was performed using ImageJ on spinal cord sections taken from the thoracic region. Co‐localization of tdTOM with OLIG2, a marker expressed throughout oligodendrocyte development, was used to distinguish tdTOM+ OPCs/NG2‐glia from other types of NG2‐expressing, tdTOM+ cell lineages such as pericytes or the vasculature. Briefly, the number of OLIG2+;tdTOM+ cells and the area (mm^2^) of the GM and WM of at least 10 thoracic hemisections were quantified per control (*Ng2‐CreER^TM^;Ascl1^F/+^;R26R^LSL‐tdTomato^*) or *Ascl1*‐CKO (*Ng2‐CreER^TM^;Ascl1^F/F^;R26R^LSL‐tdTomato^*) spinal cords at the stages indicated. The number of OLIG2+;tdTOM+/mm^2^ area of GM or WM, and percentage of OLIG2+;tdTOM+ cells showing co‐localization with the oligodendrocyte differentiation marker CC1 were determined for all the stages indicated. For mice injected with BrdU, the percentage of BrdU+;tdTOM+/total OLIG2+;tdTOM+ cells was also determined in the GM and WM of 10 thoracic hemisections of control and *Ascl1*‐CKO spinal cords. Similarly, the number of OLIG2+ cells/mm^2^ area were also determined in GM and WM of *Olig1^Cre/+^;Asc1^GFP/+^* (Control) or *Olig1^Cre/+^;Ascl1^GFP/F^* (Ascl1‐CKO) spinal cords, in which ASCL1 expression is completely lost in the oligodendrocyte lineage.

Statistical analyses were performed as previously described (Vue et al., 2014) to assess the differences in NG2‐glia cell density or percentage expression of marker+;tdTOM+ cells in GM and WM between control and *Ascl1*‐CKO. In short, a linear regression technique to model the observed cell number as a linear combination of fixed effect (genotype) and random effect (animal ID) was used. A likelihood ratio test was performed to determine the statistical significance (chi‐square, degree of freedom and *p* value) using R lmer function to conduct the linear mixed model fit by maximum likelihood. The output value of this analysis is interpreted as estimates from a traditional least squares linear regression, where the estimate for the control is the reference (or Y intercept), and the estimate for the *Ascl1*‐CKO is the value of the slope of the line from the control. These estimated output values, which are approximately the means, are reported for each genotype. A *p* < .05 indicates statistical significance that genotype (or the loss of ASCL1) accounts for the change in cell density or percentage marker+;tdTOM+ cells observed.

## 3 RESULTS

### 3.1 ASCL1 is differentially expressed in NG2‐glia in the GM and WM of the developing spinal cord

**Figure 1:**
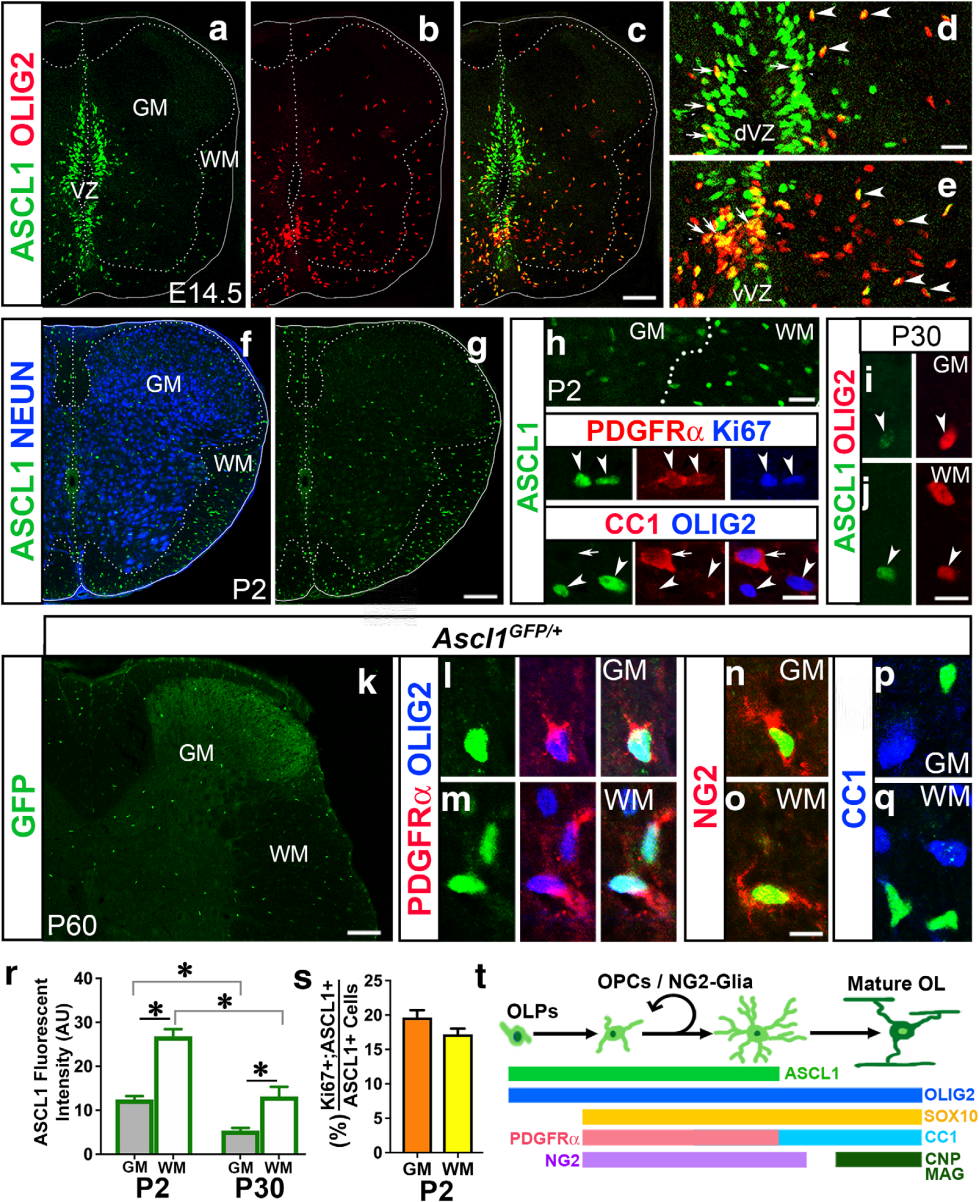
ASCL1 is expressed in OPC/NG2‐glia in the embryonic and adult spinal cord. (a–e) Immunofluorescence for ASCL1 and OLIG2 in spinal cord at E14.5. ASCL1 co‐localizes with OLIG2 in the VZ (arrows) and outside (arrowheads) of the VZ. (f–j) Immunofluorescence for ASCL1, NEUN, OLIG2, PDGFRα, Ki67, and CC1 in P2 (f–h) and P30 (i and j) spinal cords. ASCL1 expression at P2 is higher in WM than GM, and co‐localizes with OLIG2, PDGFRα, and Ki67 (arrowheads) but not with CC1 (arrow, insets). Differential expression of ASCL1 in GM and WM is maintained at P30. (k–q) Immunofluorescence for GFP in P60 *Ascl1^GFP/+^* spinal cord. GFP is expressed in PDGFRα+, OLIG2+, and NG2+ cells (l‐o) in GM and WM, but not in CC1+ cells (p and q). Dotted lines indicate GM (NEUN+) and WM boundary. Scale bar is 100 μm for a–c, f–g; 25 μm for d, e, h; and 12.5 μm for insets in h, i, j, and l–q. (r) Quantification of ASCL1 immunofluorescent intensity (AU‐arbitrary units) in OLIG2+;PDGFRα+ NG2‐glia at P2 (*N* = 3) and P30 (*N* = 2). Student's *t* test, *p < .01. Error bars are *SEM*. (s) Percentage of ASCL1+ cells that are Ki67+ at P2. (t) Overview of gene expression in NG2‐glia lineage cells [Color figure can be viewed at http://wileyonlinelibrary.com]

To investigate the function of ASCL1 in NG2‐glia, we first characterized its expression during gliogenesis in the embryonic, neonatal and adult spinal cord. As previously reported (Parras et al., 2007; Sugimori et al., 2007; Sugimori et al., 2008; Vue et al., 2014), immunofluorescence for ASCL1 showed that it is highly expressed in glial progenitors and precursor cells that span the dorsal–ventral extent of the spinal cord at E14.5 (Figure 1a). Notably, ASCL1 expression co‐localizes extensively with OLIG2 (Figure 1b,c), which at this stage marks OLPs in the dorsal and ventral ventricular zone (arrows, Figure 1d,e), as well as migrating OPCs/NG2‐glia outside of the ventricular zone (arrowheads, Figure 1d,e). By P2, ASCL1 expression in the ventricular zone is depleted but continues to be maintained in cells that have dispersed to populate the GM and WM (Figure 1f,g). Triple immunofluorescence confirmed that all the ASCL1+ cells in the GM and WM are PDGFRα+, and between 17% and 20% of these ASCL1+;PDGFRα+ are also Ki67+ (arrowheads in middle panels, Figure 1h,s). In contrast, none of the ASCL1+ cells are positive for CC1 (arrows in bottom panels, Figure 1h), a marker of immature and mature OLs. Thus, ASCL1 expression in the GM and WM in the spinal cord is limited specifically to OPCs/NG2‐glia and not differentiated OLs.

Interestingly, a fundamental difference that we observe at this neonatal stage is that ASCL1 is expressed at a higher level in OLIG2+ NG2‐glia in the WM compared wit those in the GM (top panel, Figure 1h). Indeed, by quantifying the immunofluorescent intensity of ASCL1, we found that the level of ASCL1 is twice as high in WM NG2‐glia than in GM NG2‐glia at P2 (Figure 1r). To deterhmine if ASCL1 is also differentially expressed in NG2‐glia in the GM and WM of the adult spinal cord, we performed immunofluorescence for ASCL1 at P30 (Figure 1i,j). We were able to detect ASCL1 expression in OLIG2+ cells in the WM (Figure 1j), whereas ASCL1 expression in the GM was just barely detectable in some OLIG2+ cells (Figure 1i). Quantification shows that the immunofluorescence level of ASCL1 at P30 is still twice as high in the WM compared with the GM, but overall it had decreased to about half of P2 in both the GM and WM (Figure 1r). This indicates that ASCL1 expression in NG2‐glia is not only spatially but also temporally dynamic.

To confirm if ASCL1 expression in the mature spinal cord is specific to OPC/NG2‐glia, we next performed immunofluorescence for GFP on spinal cord sections of P60 *ASCL1^GFP/+^* knock‐in mice along with OPC and OL markers. Because GFP is a stable protein, immunofluorescence for GFP should mark all ASCL1 expressing cells, including those in the GM that express very low or undetectable levels of ASCL1 at this stage. As expected, we found that GFP is expressed in cells that are sparsely scattered throughout both the GM and WM of the spinal cord (Figure 1k). Similar to the ASCL1+ cells at P2, all the GFP+ cells at this stage are OLIG2+, PDGFRα+ and NG2+ (Figure 1l–o), but were negative for CC1 (Figure 1p,q).

Collectively, these findings demonstrate that ASCL1 is present in OLPs in the ventricular zone at the onset of oligodendrogenesis, is differentially maintained in NG2‐glia in the GM and WM, and is downregulated as development proceeds from neonatal to adult stages. Furthermore, ASCL1 expression is lost when NG2‐glia transition to become CC1+ immature or mature OLs at all stages of spinal cord development (Figure 1t).

### 3.2 Progeny deriving from single ASCL1+ glial progenitor clones are restricted to GM or WM in the spinal cord

**Figure 2:**
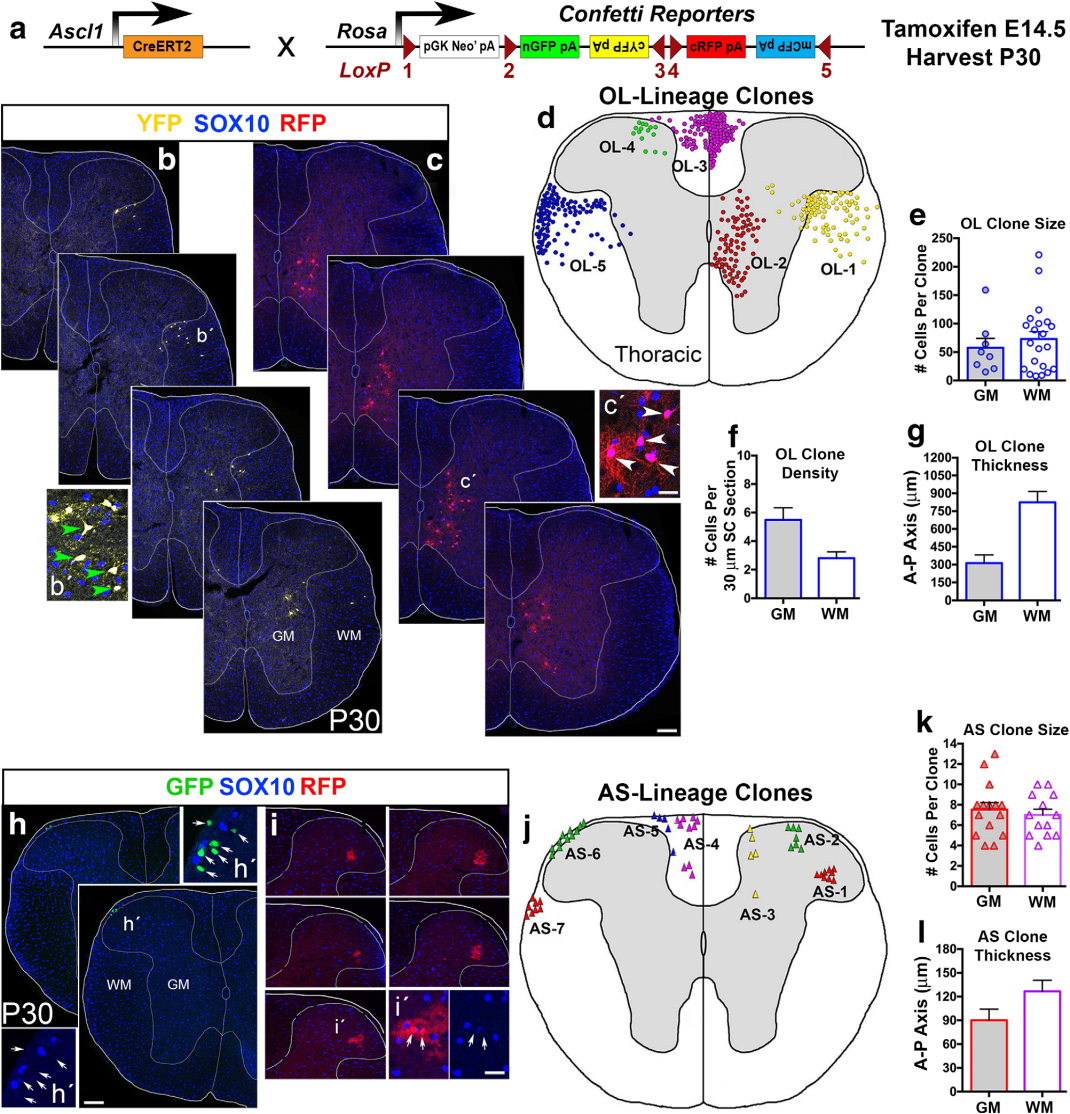
Progeny of ASCL1+ glial progenitor cells are lineage restricted to GM or WM in the spinal cord. (a) Schematic of breeding between *Ascl1^CreERT2^* and *R26R^LSL‐Confetti^* mice. Tamoxifen was administered at E14.5 and spinal cords of *Ascl1^CreERT2/+^;R26R^LSL‐Confetti/+^* mice were harvested for analysis at P30. (b and c) Examples of SOX10+;Confetti+ (YFP+ or RFP+) marked OL‐lineage clones found on serial sections of thoracic spinal cord. Arrowheads (b and c) indicate co‐localization of SOX10 with YFP and RFP, respectively. (d) Schematic composites of OL‐lineage clones forming large clusters in the spinal cord. Clones OL‐1 and OL‐2 are composites of YFP+ and RFP+ clusters from b and c, respectively. Note that cells of each OL cluster are primarily restricted to either GM or WM. (e–g) Quantification of the size, density, and thickness of OL‐lineage clones found throughout *Ascl1^CreERT2/+^;R262^LSL‐Confetti/+^* spinal cords (*N* = 5). (h and i) Composites of SOX10‐;Confetti+ (GFP or RFP) AS‐lineage clones found on adjacent sections in thoracic spinal cord. Arrows in insets (h and i) indicate that GFP+ or RFP+ cells are SOX10‐. Scale bar is 100 μm for b, c, h, i; and 25 μm for b′, c′, h′, and i′. (j) Schematic composites of AS‐lineage clones, showing restriction of each clone to either the GM or WM in the dorsal spinal cord. (k and l) Quantification of the size and thickness of AS‐lineage clones in spinal cords (*N* = 5). Error bars are *SEM* [Color figure can be viewed at http://wileyonlinelibrary.com]

Currently, it is unclear when NG2‐glia and OLs in the GM are developmentally distinct from those in the WM. Since ASCL1 is expressed in OLPs in the ventricular zone and is maintained at a higher level in WM NG2‐glia than in GM NG2‐glia, it is possible that this differential expression of ASCL1 may play a role in regulating the intrinsic properties of NG2‐glia and their distribution and development in the GM and WM of the spinal cord. We predicted that if this were the case, then clonal analysis of the developmental lineages and distribution of single ASCL1+ OLP clones should result in progeny that are spatially restricted to the GM or WM. To test this prediction, we crossed *Ascl1^CreERT2/+^* knock‐in mice (Kim et al., 2011) with the multicolor *R26R^LSL‐Confetti^* reporter mice (Snippert et al., 2010), in which stochastic Cre‐mediated recombination within the *Confetti* construct results in the low‐probability expression of either nuclear green fluorescent protein (nGFP), cytoplasmic yellow fluorescent protein (cYFP), cytoplasmic red fluorescent protein (cRFP), or membranous bound cyan fluorescent protein (mCFP) to permanently label single ASCL1+ glial progenitor clones (Figure 2a). Tamoxifen (2.5 mg/40 g body weight) was administered at E14.5 to pregnant females, and the developmental lineages and distribution of ASCL1‐derived Confetti‐labeled clones were analyzed in the spinal cords of *Ascl1^CreERT2/+^;R26R^LSL‐Confetti/+^* mice at one month of age (P30).

To ensure that we could successfully identify all Confetti‐marked clones in the spinal cord, we carefully imaged and analyzed consecutive serial sections (30 μm each) through the cervical, thoracic, and lumbar spinal cords. As reported previously for other tissues when using the *R26R^LSL‐Confetti^* mice (Schepers et al., 2012; Snippert et al., 2010), we were able to detect the fluorescence of all four Confetti reporters, each of which sparsely labeled different clusters of cells that are distributed throughout the various regions of the spinal cord (Figure 2). However, because membrane bound CFP expression is distributed extensively to cell processes, which can obscure the precise location of cell bodies or nuclei within a cluster of cells, we limited our analysis to clusters of cells that were labeled by nGFP, cYFP, or cRFP. Of the *Ascl1^CreERT2/+^;R26R^LSL‐Confetti/+^* spinal cords (*N* = 5) analyzed, we rarely observed two or more clusters of cells that were marked by the same reporter present on the same spinal cord hemisection along the anterior‐posterior (AP) axis. Because of this non‐overlapping sparse labeling by the Confetti reporters, we conclude that each cell cluster labeled by the same reporter is likely to be derived from a common ASCL1+ glial progenitor clone.

NG2‐glia are highly proliferative and have been shown to produce large clones containing hundreds of cells (Garcia‐Marques, Nunez‐Llaves, & Lopez‐Mascaraque, 2014). Similarly, we observed that the largest clones of Confetti‐marked cells were positive for the OL‐lineage specific marker SOX10 (Figure 2b,b′,c,c′). However, these SOX10+;Confetti+ clones were highly dynamic in size, ranging between 10 and 250 cells (Figure 2e). As predicted, the majority of SOX10+;Confetti+ clones were found to be restricted to either the GM or WM of *Ascl1^CreERT2/+^;R26R^LSL‐Confetti/+^* spinal cords (Figure 2d). In particular, we identified a total of 29 ASCL1‐derived SOX10+;Confetti+ clones, 21 of which were found in the WM and 8 were found in the GM (Figure 2e). A few (∼5%–10%) SOX10+;Confetti+ clones are situated at the GM/WM boundary, and contained cells distributed into both the GM and the WM; however, the distribution of these clones were typically uneven and still show strong bias in one area over the other (i.e., OL‐1 YFP+ clone, 80% WM, 20% GM, Figure 2b,d).

In agreement with previous reports that WM NG2‐glia are more proliferative than those in the GM (Dimou et al., 2008; Young et al., 2013), the average number of cells per clone was greater for the WM SOX10+;Confetti+ clones (74 cells) than those found in the GM (57 cells). Furthermore, about 40% of the WM SOX10+;Confetti+ clones were comprised of more than 100 cells per clone compared with only about 10% of SOX10+ clones in the GM (Figure 2d,e). Interestingly, the SOX10+;Confetti+ clones in the GM are more dense with regards to their distribution along the AP‐axis compared with the WM SOX10+;Confetti+ clones. For example, on average we find 5 cells per 30 μm section for the GM SOX10+;Confetti+ clones, which span across an average thickness of about 300 μm along the AP‐axis. In contrast, WM SOX10+:Confetti+ clones contain an average of 2.5 cells per 30 μm section but span an average of 900 μm thickness along the AP‐axis (Figure 2f,g). Therefore, WM NG2‐glia not only generate a larger clone of cells but also spread more widely to occupy a thicker volume.

In addition to SOX10+ OL‐lineage clones, we identified SOX10‐ clones that are also spatially restricted in the GM or WM in the dorsal spinal cord (arrows, Figure 2h–j). These SOX10‐ clones are AS based on their morphology and co‐localization with GFAP (not shown), and are likely derived from the ASCL1+;OLIG2‐ AS progenitors in the ventricular zone of the dorsal spinal cord, as we have previously demonstrated (Vue et al., 2014). Unlike, the SOX10+ OL‐lineage clones, these AS‐lineage clones contained on average of only 8 cells per clone in both the GM and WM, and occupied an AP‐thickness of about 90–120 μm (Figure 2k,l).

### 3.3 ASCL1 is efficiently knocked out in NG2‐glia in the spinal cord

ASCL1 is required for maintaining the neural progenitor pool during development as well as in areas of adult neurogenesis such as the subventricular zone (SVZ) of the lateral ventricle and the subgranular zone (SGZ) of the hippocampal dentate gyrus (Andersen et al., 2014; Kim et al., 2011). Therefore, it is not surprising that ASCL1 is expressed in NG2‐glia in both the developing and adult CNS. However, the precise requirement of ASCL1 specifically in NG2‐glia at these different stages remains unclear. To address this, we used *Ng2‐CreER^TM^* to conditionally delete *Ascl1^Floxed^* alleles (Pacary et al., 2011) in embryonic NG2‐glia at E14.5 or in the adult at P30. These *Ng2‐CreER^TM^* mice are BAC transgenics that express a tamoxifen‐inducible Cre recombinase under the control of the mouse NG2 (*Cspg4*) promoter/enhancer (Zhu et al., 2011).

**Figure 3:**
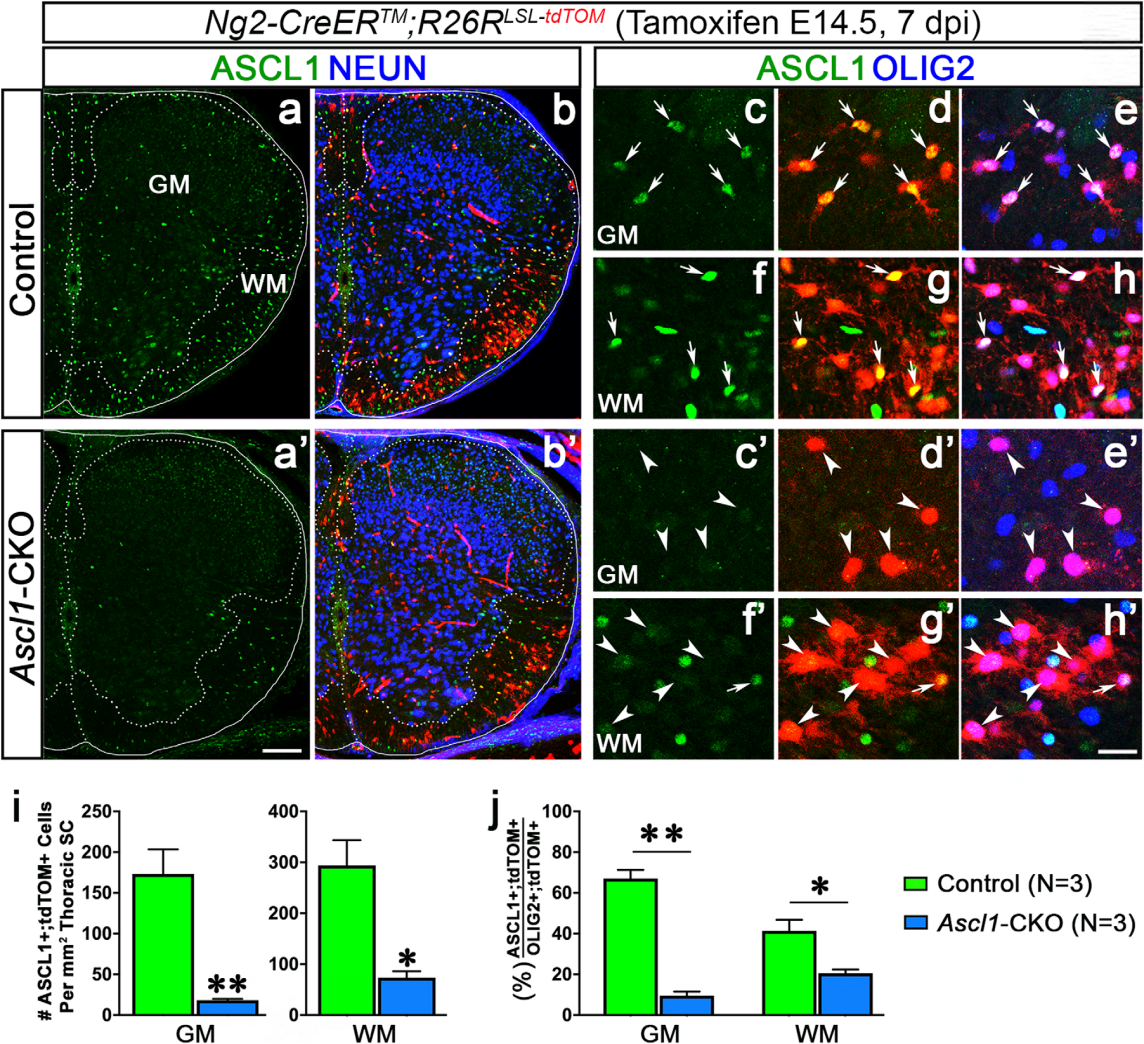
*Ascl1^Floxed^* allele is efficiently deleted in NG2‐glia. Immunofluorescence for ASCL1 in spinal cords of control (a–h) versus Ascl1‐CKO (a′–h′) *Ng2‐CreER^TM^;R26R^LSL‐tdTOM^* mice exposed to tamoxifen at E14.5 and harvested 7 dpi at P2. (a–h) ASCL1 is expressed in most of the OLIG2+;tdTOM+ cells in the GM (arrows, c–e) and WM (arrows, f–h) of control spinal cord. (a′–h′) ASCL1 expression is lost in OLIG2+;tdTOM+ cells in the GM (arrowheads, c′–e′) and WM (arrowheads, f′–h′) of *Ascl1*‐CKO spinal cord. Scale bar is 100 μm for a and b, a′ and b′; and 25 μm for c–h and c′–h′. (i and j) Quantification of the density of ASCL1+;tdTOM+ cells/mm^2^ in the GM and WM of 30 μm thoracic hemisection, and the percentage of ASCL1+;tdTOM+/total OLIG2+;tdTOM+ cells in spinal cords of control versus *Ascl1*‐CKO mice. *p < .01, **p < .001. Error bars are *SEM* [Color figure can be viewed at http://wileyonlinelibrary.com]

We first examined the efficiency of *Ascl1* deletion in tdTOM‐labeled NG2‐glia by analyzing expression of ASCL1 in neonatal spinal cords of *Ng2‐CreER^TM^;Ascl1^F/+^;R26R^LSL‐tdTOM^* mice (Control) in comparison to *Ng2‐CreER^TM^;Ascl1^F/F^;R26R^LSL‐tdTOM^* (*Ascl1*‐CKO) littermates exposed to tamoxifen at E14.5. Immunofluorescence for ASCL1 seven days post‐tamoxifen induction (7 dpi) at P2 showed that there was a significant decrease in the total number of ASCL1+ expressing cells in both the GM and WM of the *Ascl1*‐CKO spinal cords in comparison to controls (Figure 3a vs. a′). The decrease in ASCL1 expression was specific to tdTOM+ cells in the *Ascl1*‐CKO spinal cords (arrowheads, Figure 3c′–h′), whereas ASCL1 expression was still maintained in the majority of OLIG2+;tdTOM+ cells in the control spinal cords (arrows, Figure 3c–h). Notably, expression of SOX10 (not shown) and OLIG2 were unaffected in tdTOM+ cells of the *Ascl1*‐CKO at 7 dpi (Figure 3e′,h′), demonstrating that the loss of ASCL1 did not alter the fate of the NG2‐glia away from the OL‐lineage.

We then quantified and compared the number of ASCL1+;tdTOM+ cells in the GM and WM of control (*N* = 3) versus *Ascl1*‐CKO (*N* = 3) thoracic spinal cords (∼10 hemisections/Th‐SC). We observed about 173 ± 30 ASCL1+;tdTOM+ cells/mm^2^ in the GM and 294 ± 50 ASCL1+;tdTOM+ cells/mm^2^ in the WM of control spinal cords (green bars, Figure 3i). These numbers represent 67% and 41% of the total number of OLIG2+;tdTOM+ cells in the GM and WM, respectively (green bars, Figure 3j). Within the *Ascl1*‐CKO, the number of ASCL1+;tdTOM+ cells was reduced significantly to just 18 ± 2 cells/mm^2^ in the GM and 73 ± 13 cells/mm^2^ in the WM, which represent about 10% and 21% of the total number of OLIG2+;tdTOM+ cells (blue bars, Figure 3i,j). This dramatic decrease in the number and proportion of ASCL1+;tdTOM+ cells in the *Ascl1*‐CKO is indicative that *Ng2‐CreER^TM^* was efficient in deleting *Ascl1^Floxed^* alleles in NG2‐glia in the spinal cord.

### 3.4 ASCL1 is required for regulating the number but not differentiation of embryonic OPCs in the spinal cord

**Figure 4:**
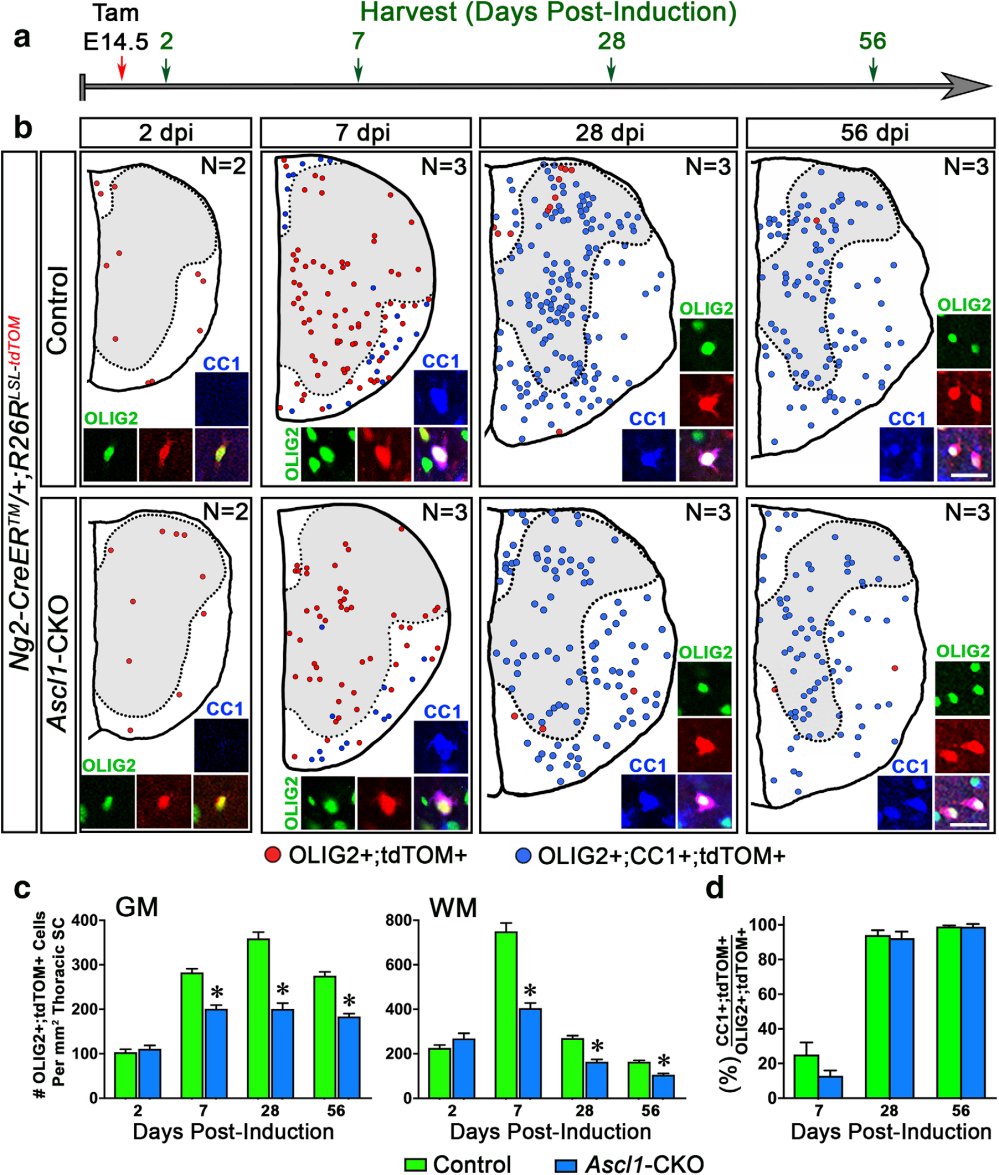
*Ascl1*‐CKO at E14.5 decreases the number of NG2‐lineage cells in the developing spinal cord. (a) Schematic of tamoxifen induction at E14.5 and days of spinal cord harvest of *Ng2‐CreER^TM^;R26R^LSL‐tdTOM^* mice. (b) Schematic of representative thoracic spinal cord hemisections showing OLIG2+;tdTOM+ (red dots) and OLIG2+;CC1+;tdTOM+ (blue dots) in the GM and WM of the control versus *Ascl1*‐CKO spinal cords at 2, 7, 28, and 56 dpi. Insets are immunofluorescence showing co‐localization of OLIG2 and CC1 with tdTOM. Number of spinal cords quantified for each stage per genotype is indicated. Scale bar for insets is 25 μm. (c and d) Quantification of the density of OLIG2+;tdTOM+ cells/mm^2^ in the GM and WM of 30 μm thoracic hemisection, and the percentage of OLIG2+;CC1+;tdTOM+/total OLIG2+;tdTOM+ cells in the control versus *Ascl1*‐CKO spinal cords. *p < .01. Error bars are *SEM* [Color figure can be viewed at http://wileyonlinelibrary.com]

To fully appreciate the role of ASCL1 specifically in NG2‐glia, we next examined the developmental dynamics of embryonic NG2‐glia over time by quantifying the number of OLIG2+;tdTOM+ cells/mm^2^ area in both the GM and WM of control (*Ng2‐CreER^TM^;Ascl1^F/+^;R26R^LSL‐tdTOM^*) and *Ascl1*‐CKO (*Ng2‐CreER^TM^;Ascl1^F/F^;R26R^LSL‐tdTOM^*) spinal cords from mice given tamoxifen at E14.5, and analyzed at 2, 7, 28, and 56 dpi (Figure 4a–c). Because NG2 is also expressed in pericytes, some of which are also labeled by tdTOM, the quantification of OLIG2+;tdTOM+ cells (insets, Figure 4b) ensures that only NG2‐glia derived tdTOM+ cells were being counted.

In control spinal cords (top panels, Figure 4b), at 2 dpi there were 105 ± 18 and 226 ± 38 OLIG2+;tdTOM+ cells/mm^2^ in the GM and WM, respectively. By 7 dpi, this number had increased about 2.7‐fold to 283 ± 27 in the GM and 3.3‐fold to 751 ± 119 cells in the WM. From 7 to 28 dpi, the number of OLIG2+;tdTOM+ cells/mm^2^ underwent a slight increase in the GM to 359 ± 47 but was decreased in the WM to 270 ± 36. This decreased in the WM is likely attributed to the fact that we are quantifying from fixed 30 μm spinal cord sections, but the area of the WM is increased by more than three‐fold from 7 to 28 dpi (not shown), in addition to a significant expansion in the length of the spinal cord. Consequently, this growth of the spinal cord would result in a spreading or thinning of OLIG2+;tdTOM+ cells/mm^2^ area in the WM. By 56 dpi, we observed that there was an overall decrease in the number of OLIG2+;tdTOM+ cells/mm^2^ in both the GM (275 ± 30) and WM (164 ± 20; green bars, Fig. 4C) compared with at 28 dpi. Given that the growth of the spinal cord from 28 to 56 dpi is not very drastic, it is possible that this decrease may reflect a turnover of OLIG2+;tdTOM+ cells.

Overall, these findings demonstrate that embryonic NG2‐glia labeled as a population at E14.5 in control spinal cords proliferate dramatically up until the first postnatal week, then their rate of proliferation decreases significantly thereafter and may completely cease by about a month of age. Accordingly, when we quantified the proportion of OLIG2+;tdTOM+ cells that had expressed the OL marker CC1 (CC1+;tdTOM+; blue cells, Figure 4b), we found that none of the OLIG2+;tdTOM+ were CC1+ at 2 dpi, whereas around 25% were CC1+ at 7 dpi. Thus, the majority of OLIG2+;tdTOM+ cells within the first postnatal week are proliferating OPCs. However, by 28 dpi, nearly all (>95%) of the OLIG2+;tdTOM+ cells had become CC1+ (green bars, Figure 4d), indicating a differentiation into OLs, which clearly explains the lack of increase in OLIG2+;tdTOM+ cells from 28 to 56 dpi.

Within *Ascl1*‐CKO spinal cords (bottom panels, Figure 4b), we observed a similar initial number of OLIG2+;tdTOM+ cells/mm^2^ in the GM (110 ± 23) and WM (266 ± 69) as seen in the control at 2 dpi (blue bars, Figure 4c). This was expected since the initial number of OLIG2+;tdTOM+ cells/mm^2^ should not be affected by *Ascl1*‐CKO. By 7 dpi, the number of OLIG2+;tdTOM+ cells/mm^2^ in the GM (201 ± 28) and WM (404 ± 76) of *Ascl1*‐CKO spinal cords increased compared with at 2 dpi (blue bars, Figure 4c), but this increase was less than the increase seen in the control. Furthermore, the number of OLIG2+;tdTOM+ cells/mm^2^ in the *Ascl1*‐CKO spinal cords remained unchanged from 28 and 56 dpi in the GM, which was unlike in the control, and was consistently lower in the WM compared with the WM of control (blue bars, Figure 4c). Overall, the decrease in OLIG2+;tdTOM+ cells/mm^2^ in the *Ascl1*‐CKO was significant in both the GM and WM at 7, 28, and 56 dpi in comparison to control spinal cords, indicating that the proliferation of embryonic NG2‐glia is compromised in the absence of ASCL1.

To examine if the loss of ASCL1 affected the differentiation of embryonic NG2‐glia into OL, we then quantified the percentage of OLIG2+;tdTOM+ cells that also expressed CC1 in *Ascl1*‐CKO spinal cords (Figure 4b,d). At 2 dpi, as in the control we did not observe any CC1+;tdTOM+ cells in *Ascl1*‐CKO spinal cords. At 7 dpi, the percentage of CC1+;tdTOM+ cells was lower in *Ascl1*‐CKO (∼13%) spinal cords compared with control (∼25%), indicating a possible delay in differentiation, although this difference was not enough to be significant (*p* < .08). From 28 to 56 dpi, greater than 95% of the OLIG2+;tdTOM+ cells had also differentiated into CC1+ OL in the *Ascl1*‐CKO spinal cords as seen for the control (blue bars, Figure 4d). Additionally, expression of mature OL markers such as myelin‐associated glycoprotein (MAG) and 2′,3′‐cyclic nucleotide 3′‐phosphodiesterase (CNP) were also not affected in OLIG2+;tdTOM+ cells of *Ascl1*‐CKO spinal cords (arrows, Figure 6b,d). These findings illustrate that the loss of ASCL1 did not impede embryonic NG2‐glia from differentiating in mature OLs.

Taken together, these results show that during oligodendrogenesis in the spinal cord, ASCL1 is required to ensure a proper generation of the number of NG2‐glia but not their differentiation into mature OL.

### 3.5 Ascl1‐CKO decreases the proliferation of adult NG2‐glia in the spinal cord

**Figure 5:**
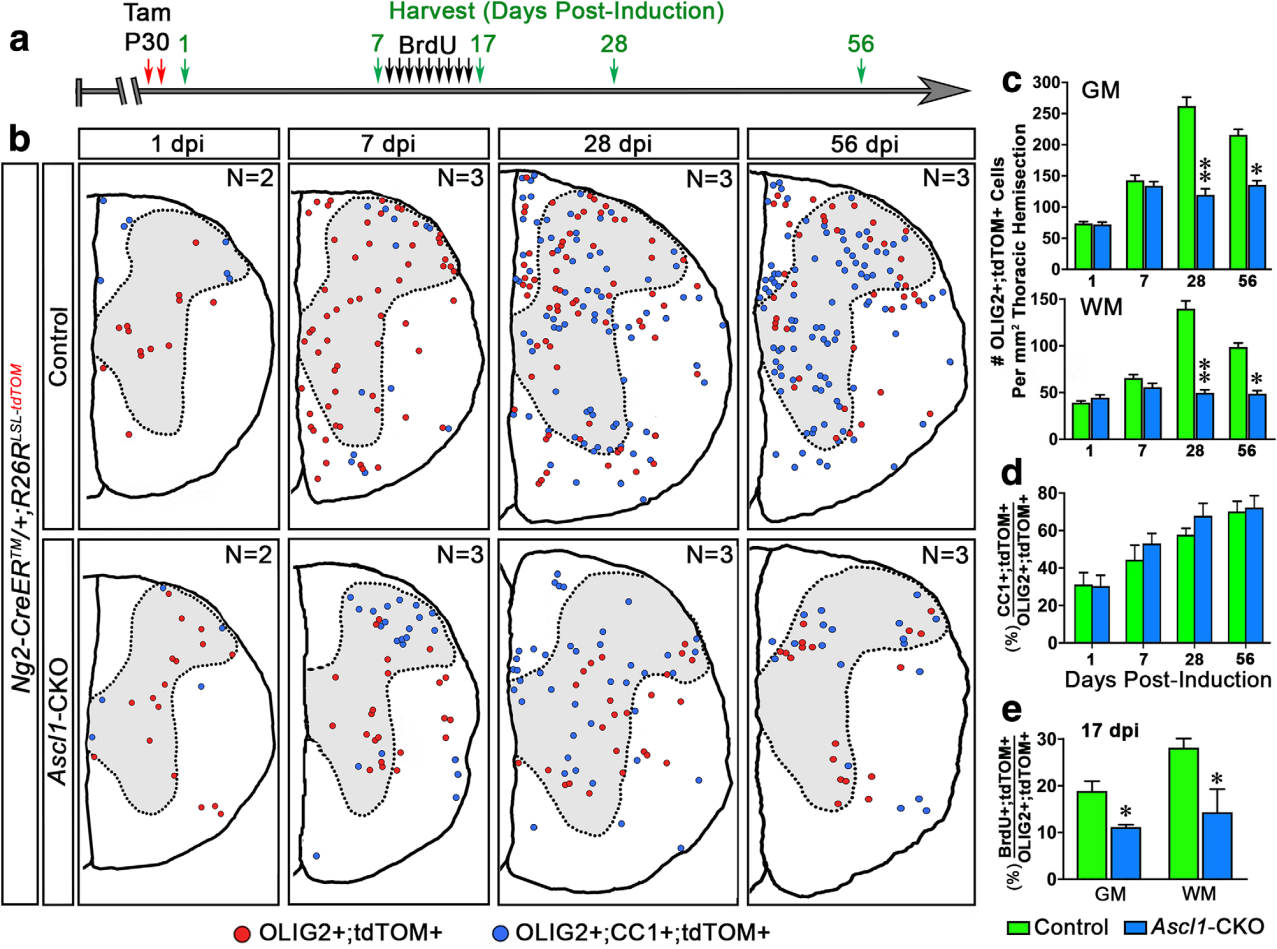
*Ascl1*‐CKO at P30 decreases the proliferation of NG2‐glia in the adult spinal cord. (a) Schematic of tamoxifen induction and BrdU administration of adult *Ng2‐CreER^TM^;R26R^LSL‐tdTOM^* mice and days of spinal cord harvest post‐induction. (b) Schematic of representative thoracic spinal cord hemisections showing OLIG2+;tdTOM+ (red dots) and OLIG2+;CC1+;tdTOM+ (blue dots) of NG2‐lineage cells in the GM and WM of control versus *Ascl1*‐CKO spinal cords at 1, 7, 28, and 56 dpi. Number of spinal cords quantified for each stage per genotype is indicated. (c and d) Quantification of the number of OLIG2+;tdTOM+/mm^2^ area in the GM and WM of 30 μm thoracic hemisection, and the percentage of OLIG2+;CC1+;tdTOM+/total OLIG2+;tdTOM+ cells in the control versus *Ascl1*‐CKO spinal cords. (e) Quantification of the percentage of BrdU+;tdTOM+/total OLIG2+;tdTOM+ cells in control versus *Ascl1*‐CKO spinal cords at 17 dpi. *p < .01. **p < .001. Error bars are *SEM* [Color figure can be viewed at http://wileyonlinelibrary.com]

To determine if ASCL1 is also required for regulating the development of NG2‐glia in the adult CNS, we analyzed the spinal cords of *Ascl1‐*CKO and control littermates that were administered with two doses of tamoxifen (2.5 mg/40 g body weight) over two days starting at P30 (red arrows, Figure 5a). The number of OLIG2+;tdTOM+ cells/mm^2^ in both the GM and WM of thoracic spinal cord hemisections at 1, 7, 28, and 56 dpi was determined (Figure 5b,c).

Within control spinal cords (top panels, Figure 5b), at 1 dpi there were 74 ± 11 and 39 ± 6 OLIG2+;tdTOM+ cells/mm^2^ in GM and WM, respectively. This number then doubled to 143 ± 28 in GM and 66 ± 12 in WM by 7 dpi, and more than tripled to 262 ± 47 in the GM and 139 ± 10 in the WM by 28 dpi. By 56 dpi, the number of OLIG2+;tdTOM+ cells/mm^2^ was decreased to 216 ± 30 in the GM and 99 ± 15 in the WM, suggesting that adult NG2‐glia labeled at P30 may also start to turnover between 28 and 56 dpi (green bars, Figure 5c).

Within *Ascl1*‐CKO spinal cords, the initial number of OLIG2+;tdTOM+ cells/mm^2^ in the GM (72 ± 12) and WM (45 ± 11) at 1 dpi was similar to control. In contrast, although the number of OLIG2+;tdTOM+ cells/mm^2^ increased to 135 ± 21 in GM and 56 ± 14 in WM by 7 dpi in *Ascl1*‐CKO spinal cords, this number remained relatively constant at 28 dpi (119 ± 30 in GM, 50 ± 10 in WM) as well as at 56 dpi (136 ± 22 in GM, 49 ± 11 in WM; blue bars, Figure 5c), which was unlike what was observed for the control spinal cords. This lack of increase in the number of OLIG2+;tdTOM+ cells/mm^2^ in the *Ascl1*‐CKO was statistically significant at 28 and 56 dpi, and implies that the proliferation of adult NG2‐glia was severely compromised in the absence of ASCL1.

To investigate if the failure for adult NG2‐glia to increase after 7 dpi in the *Ascl1*‐CKO was indeed due to a reduction of cell proliferation, we used bromodeoxyuridine (BrdU), a thymidine analog that is incorporated into newly synthesized DNA, to specifically label replicating cells. Control and *Ascl1*‐CKO littermate mice were administered with two pulses of tamoxifen starting at P30, and BrdU (3 mg/25 g body weight) was then injected once per day for 10 consecutive days starting at 8–17 dpi (black arrows, Figure 5a). We observed that there was far fewer BrdU+;tdTOM+ cells in thoracic spinal cord at 17 dpi in the *Ascl1*‐CKO compared with controls. Indeed, by analyzing the proportion of BrdU+;tdTOM+/total OLIG2+;tdTOM+ cells, we found that the percentage of proliferating cells in the control was around 18.5% in the GM and 28.1% in the WM (green bars, Figure 5e), and was significantly reduced in the *Ascl1*‐CKO spinal cords to 11.4% in the GM and 14.4% in the WM (blue bars, Figure 5e). This finding strongly suggests that the decrease in the overall number of OLIG2+;tdTOM+/mm^2^ cells in *Ascl1*‐CKO spinal cords is likely due to a decrease in the ability of adult NG2‐glia to proliferate in the absence of ASCL1.

**Figure 6:**
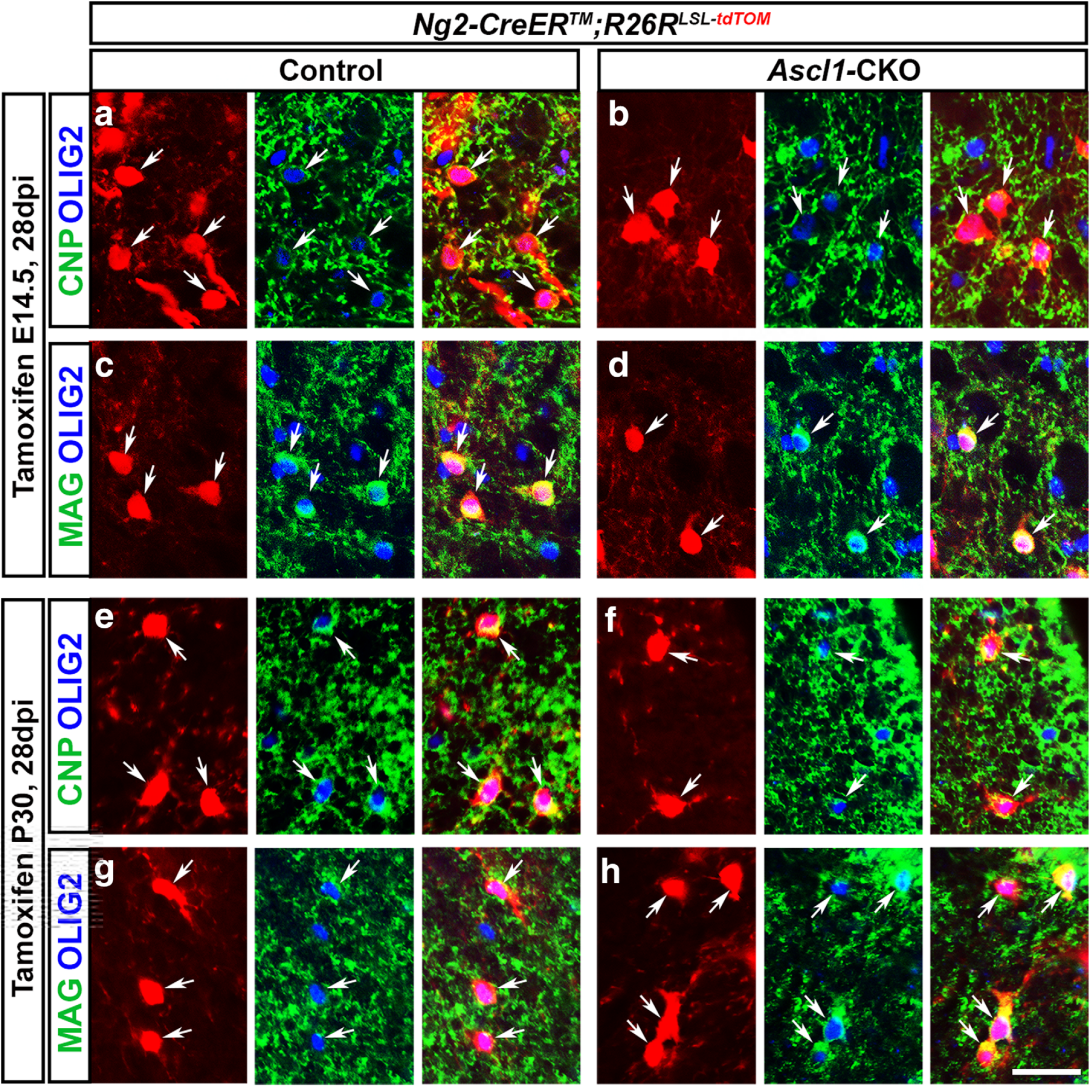
Expression of mature oligodendrocyte markers are unaffected in *Ascl1*‐CKO NG2‐glia. (a–h) Immunofluorescence for OLIG2 with CNP or MAG in control or *Ascl1*‐CKO *Ng2‐CreER^TM^;R26R^LSL‐tdTOM^* spinal cords tamoxifen at E14.5 (a–d) or P30 (e–h) and harvest at 28 dpi. CNP and MAG co‐labeled with OLIG2+;tdTOM+ cells (arrows) of *Ascl1*‐CKO (c, d, g, and h) as in control (a, b, e, and f). Scale bar is 25 μm [Color figure can be viewed at http://wileyonlinelibrary.com]

As in embryonic spinal cord development, we did not observe any significant difference in the percentage of CC1+;tdTOM+ cells between the control and *Ascl1*‐CKO spinal cords at 1, 7, 28, or 56 dpi following tamoxifen administration at P30 (Figure 5d). Furthermore, both MAG and CNP were also expressed in OLIG2+;tdTOM+ cells of *Ascl1*‐CKO (Figure 6f,h) as in the control (Figure 6e,g). The expression of these myelinating OL markers argues that the ability of adult NG2‐glia to differentiate was not affected by the loss of ASCL1. However, a notable difference between OLIG2+;tdTOM+ cells that were labeled by *Ng2‐CreER^TM^* at P30 from those labeled at E14.5 is that around 30% of the cells were already CC1+ at 1 dpi (Figure 5b,c), compared with 0% at E14.5. Furthermore, whereas more than 95% of OLIG2+;tdTOM+ cells labeled at E14.5 had differentiated to become CC1+ by 28 dpi (Figure 4d), only about 70% of OLIG2+;tdTOM+ cells labeled at P30 were CC1+, even at 56 dpi (Figure 5d). This indicates that adult NG2‐glia proliferate and differentiate at a much slower pace than embryonic NG2‐glia in the spinal cord, and about 30% of the adult NG2‐glia, as labeled by *Ng2‐CreER^TM^*, may continue to remain undifferentiated on a long‐term basis.

### 3.6 The size of NG2‐glia clones is decreased in the absence of ASCL1

**Figure 7:**
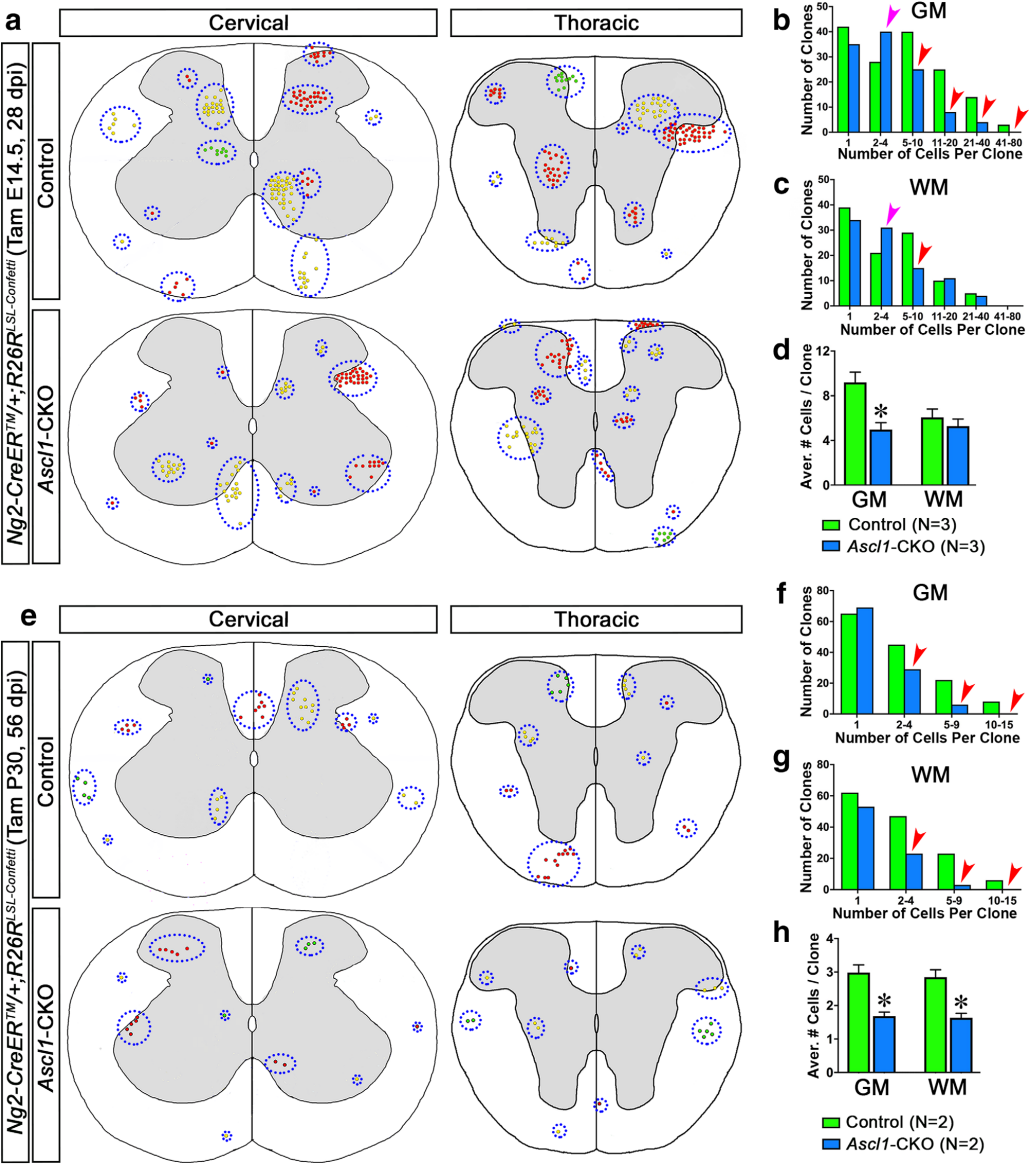
*Ascl1*‐CKO decreases the size of NG2 clones in spinal cord. (a and e) Schematic composites of nGFP, cRFP, or cYFP+ NG2‐glia lineage clones found in the cervical and thoracic spinal cords of *Ng2‐CreER^TM^;R262^LSL‐Confetti^* mice labeled at E14.5 or P30. Dotted ovals indicate Confetti+ cells belonging to the same control or *Ascl1*‐CKO clone found on adjacent spinal cord sections. (b–d, f–h) Quantification of the number of clones based on clone size and the average number of cells per clone in the GM and WM of control and *Ascl1‐*CKO NG2‐glia clones. Pink and red arrowheads indicate increased and decreased number of clones for a particular size clone, respectively. *p < .01. Error bars are *SEM* [Color figure can be viewed at http://wileyonlinelibrary.com]

To better contrast the developmental dynamics of embryonic and adult NG2‐glia, and how the loss of ASCL1 affected the development of these cells at the clonal level, we next incorporated the *R26R^LSL‐Confetti^* allele into *Ng2‐CreER^TM^;Ascl1^Floxed^* mice. By administering tamoxifen to mice harboring a combination of all three alleles, we then traced the developmental lineage of single NG2‐glia clones in the presence or absence of ASCL1 in the spinal cord. Tamoxifen (0.625 mg/40 g body weight) was administered to pregnant females at E14.5 to sparsely label control (*Ng2‐CreER^TM^;Ascl1^F/+^;R26R^LSL‐Confetti^*) or *Ascl1*‐CKO (*Ng2‐CreER^TM^;Ascl1^F/F^;R26R^LSL‐Confetti^*) NG2‐glia clones in the spinal cords of embryos. Spinal cords were harvested and analyzed for Confetti+ cells at 28 dpi, at which point greater than 95% of the labeled cells should have differentiated to become CC1+ OL, as indicated earlier by tdTOM labeling (Figure 4b,d). Similar to the *Ascl1^CreERT2/+^;R26R^LSL‐Confetti/+^* mice (Figure 2), we found clusters of SOX10+;Confetti+ or OLIG2+;Confetti+ cells sparsely distributed along serial sections of the cervical, thoracic and lumbar spinal cords of both *Ng2‐CreER^TM^;Ascl1^F/+^;R26R^LSL‐Confetti^* and *Ng2‐CreER^TM^;Ascl1^F/F^;R26R^LSL‐Confetti^* mice, where each cluster is labeled by the same reporter (nGFP, cRFP, or cYFP), and thus is likely to be derived from a common NG2‐glia clone (Figure 7a).

Through extensive analysis of serial sections from multiple spinal cords (*N* = 3) of *Ng2‐CreER^TM^;Ascl1^F/+^;R26R^LSL‐Confetti^* mice, we identified more than 250 control NG2‐glia derived Confetti+ clones in the GM and WM, altogether containing about 1,900 labeled cells. About 30% of these clones consist of just a single cell, 60% contained between 2 and 20 cells per clone, while the remaining 10% contained between 20 and 80 cells per clone (green bars, Figure 7a–c). This finding demonstrates that the majority of NG2‐glia clones, even when labeled at E14.5, do not proliferate at all or proliferate only a few times to give rise to between 1 and 20 cells per clone. Indeed, when we quantified the average number of cells per clone, there was 9.2 ± 0.9 cells per GM Confetti+ clone and 6.1 ± 0.8 cells per WM Confetti+ clone in *Ng2‐CreER^TM^;R26R^LSL‐Confetti^* spinal cords (green bars, Figure 7d). This average was a 5 to10 fold decrease compared with the Confetti+ clones in the GM and WM of *Ascl1^CreERT2/+^;R26R^LSL‐Confetti/+^* spinal cords, in which 80% of the clones contained between 20 and 250 cells per clone (Figure 2d,e), indicating that *Ascl1^CreERT2^* and *Ng2‐CreER^TM^* label temporally distinct OLP or NG2‐glia populations.

We next analyzed E14.5 *Ascl1*‐CKO NG2‐glia clones from a similar volume of serial sections of spinal cords (*N* = 3) from *Ng2‐CreER^TM^;Ascl1^F/F^;R26R^LSL‐Confetti^* mice at 28 dpi. We identified about 200 *Ascl1*‐CKO Confetti+ clones, containing a total of about 1,140 labeled cells (blue bars, Figure 7b,c), which are dramatically fewer than the overall number of control clones labeled in *Ng2‐CreER^TM^;Ascl1^F/+^;R26R^LSL‐Confetti^* spinal cords. This decrease was not completely unexpected since the number of NG2‐glia lineage cells at the population level, as labeled by tdTOM, was also decreased with *Ascl1‐*CKO (Figure 4b,c). A notable feature that we frequently observed was that *Ascl1*‐CKO Confetti+ clones contained fewer cells per clone than the control Confetti+ clones (Figure 7a). In particular, when we evaluated the number of *Ascl1*‐CKO Confetti+ clones based on clone size, we found that in the GM there was an increase in clone numbers for those that contained between 2 and 4 cells per clone (pink arrowhead, Figure 7b), along with a concomitant decrease in clone numbers for those that contained more than 5 cells per clone (red arrowheads, Figure 7b). Similarly, *Ascl1*‐CKO Confetti clones in the WM also showed an increased number of clones containing 2–4 cells per clone (pink arrowhead, Figure 7c), and a concomitant decrease in clones containing between 5 and 10 cells per clone (red arrowhead, Figure 7c). Interestingly, there was no decrease in the number of clones in WM *Ascl1*‐CKO Confetti mice that contained more than 10 cells per clone compared with the control (Figure 7c). This suggests that *Ascl1*‐CKO may not negatively affect the proliferation of highly proliferative NG2‐glia in the WM, or that these cells may have escaped *Ascl1*‐CKO altogether. Indeed, when we quantified the overall average number of cells per clone, GM but not WM *Ascl1‐*CKO Confetti+ clones exhibit a 46% decrease (5.0 ± 0.6 GM and 5.3 ± 0.6 WM; blue bars, Figure 7d), in comparison to their respective controls (9.2 ± 0.9 GM, and 6.1 ± 0.8 WM; green bars, Figure 7d).

To further assess if *Ascl1‐*CKO has a similar effect on the development of NG2 clones in the adult spinal cord, we administered a single dose of tamoxifen (0.625 mg/40 g body weight) at P30 to *Ng2‐CreER^TM^;Ascl1^F/+^;R26R^LSL‐Confetti^* or *Ng2‐CreER^TM^;Ascl1^F/F^;R26R^LSL‐Confetti^* mice. Equal number (*N* = 2) and volume of spinal cords were serially sectioned and analyzed for Confetti+ clones at 56 dpi for each genotype (Figure 7e). We identified about 280 control Confetti+ clones in *Ng2‐CreER^TM^;Ascl1^F/+^;R26R^LSL‐Confetti^* spinal cords, which comprised a total of about 800 labeled cells that were similarly distributed into the GM and WM. About 45% of these clones were single cell clones, 50% were clones that contained between 2 and 9 cells, while the remaining 5% were larger clones containing between 10 and 15 cells (green bars, Figure 7f,g). Quantification revealed that the average number of cells per clone for these P30 control NG2‐glia clones was 3.0 ± 0.2 in the GM and 2.8 ± 0.2 in the WM (green bars, Figure 7h), which was noticeably lower than the embryonic NG2 clones labeled at E14.5 (green bars, Figure 7d).

In contrast, we identified only about 180 *Ascl1‐*CKO Confetti+ clones in both the GM and WM of *Ng2‐CreER^TM^;Ascl1^F/F^;R26R^LSL‐Confetti^* spinal cords, comprising around 300 total labeled cells. This dramatic decrease in the overall number of *Ascl1‐*CKO Confetti+ clones suggests that there may be an increase in NG2‐glia death or turnover in the absence of ASCL1. Interestingly, this decrease was only noted for *Ascl1‐*CKO Confetti+ clones that contained at least 2 cells per clone but not for single cell clones (red arrowheads, Figure 7f,g), further supporting the notion that only proliferating NG2‐glia are affected by the loss of ASCL1. Quantification revealed that the average number of cells per clone for *Ascl1‐*CKO clones (blue bars, Figure 7h) was reduced by nearly half (1.7 ± 0.1 in the GM and 1.6 ± 0.1 in the WM) compared with control clones (green bars, Figure 7h).

Collectively, these findings demonstrate that at the clonal level, the proliferation of NG2‐glia is highly dynamic, and on average is reduced by two to three‐fold as development proceeds from the embryo into the adult. Furthermore, ASCL1 seems to play a vital role in shaping the survival as well as the proliferation of NG2‐glia, as the number of Confetti‐labeled NG2‐glia clones is reduced with *Ascl1‐*CKO and the size of NG2‐glia clones seems to favor the generation of smaller clones at the expense of larger highly proliferative NG2‐glia clones.

### 3.7 Complete deletion of Ascl1 with Olig1^Cre^ decreases the number but not differentiation of OLIG2+ cells in the spinal cord

A limitation with using Ng2‐CreER^TM^ is that some OLIG2+;tdTOM+ cells escaped *Ascl1^Floxed^* allele deletion (Figure 3a′–h′). Although these cells comprised of only a small portion (∼10%) of the overall OLIG2+;tdTOM+ cells, it is possible that they could potentially masked the proliferation and differentiation phenotype of the surrounding *Ascl1*‐CKO OLIG2+;tdTOM+ cells over time. To overcome this, and to determine the absolute requirement of ASCL1 in the generation and development of NG2‐glia, we crossed *Olig1^Cre/+^;Ascl1^GFP/+^* knock‐in mice with *Ascl1^F/+^* mice. Because Cre‐recombinase is constitutively expressed at the onset of oligodendrogenesis under the endogenous *Olig1* locus, this cross ensures efficient deletion of *Ascl1^Floxed^* allele in all OLPs in the ventricular zone of *Olig1^Cre/+^;Ascl1^GFP/F^* (*Ascl1*‐CKO) even prior to the generation of OPCs/NG2‐glia.

**Figure 8:**
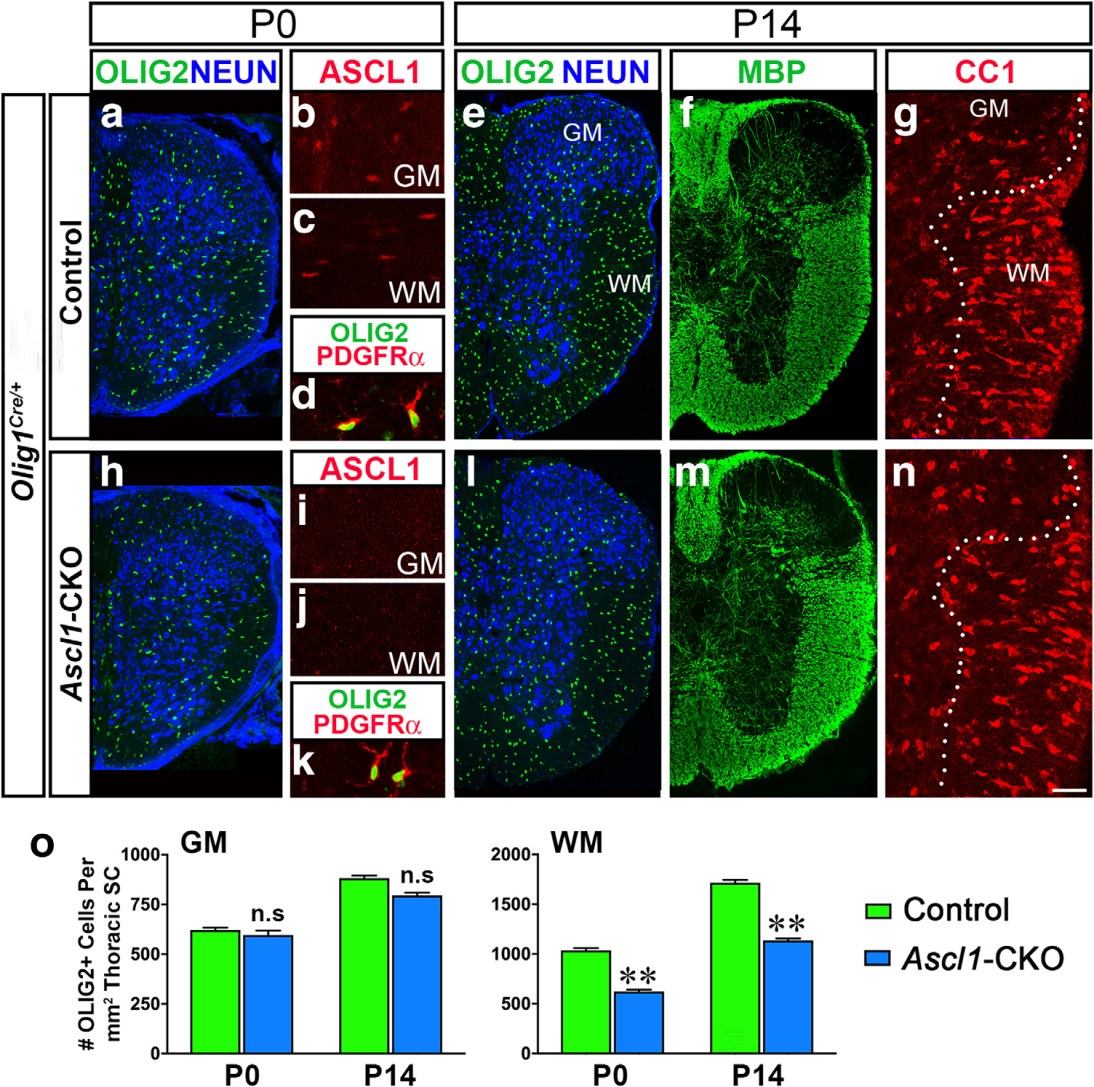
*Ascl1*‐CKO decreases generation of OLIG2+ cells but not differentiation of oligodendrocytes. (a–n) Immunohistochemistry of *Olig1^Cre/+^*;Control (a–g) or *Olig1^Cre/+^*;*Ascl1*‐CKO (h–n) spinal cords at P0 or P14. ASCL1 is no longer expressed in NG2‐glia in the GM and WM of *Ascl1*‐CKO (i and j) as in control (b and c). Number of OLIG2+ cells is noticeably decreased in WM of *Ascl1*‐CKO at P0 and P14, but expression of PDGFRα (k), MBP (m) and CC1 (n) are unaffected and similar to control (d, f, and g). NEUN marks GM and dotted lines in F and L indicated GM/WM boundary. Scale bar is 100 μm for a, e, f, h, l, and m; and 25 μm for b, c, d, g, i, j, k, and n. (o) Quantification of the density of OLIG2+ cells/mm^2^ in the GM and WM of 30 μm thoracic hemisection. N=4 hemisections/genotype for P0 and P14 spinal cords. *p < .01. Error bars are *SEM* [Color figure can be viewed at http://wileyonlinelibrary.com]

As expected, immunofluorescence of spinal cord sections at E15.5 (not shown) and P0 demonstrated that ASCL1 expression was completely absent in the GM and WM of *Ascl1*‐CKO (Figure 8i,j) but not in control (Figure 8b,c). As seen in germline *Ascl1* mutants (Sugimori et al., 2007; Sugimori et al., 2008), the loss of ASCL1 did not compromise the generation of NG2‐glia, as marked by OLIG2 and PDGFRα in the *Ascl1*‐CKO (Figure 8d,k), but there was a noticeable decrease in the number of OLIG2+ cells in the WM at P0 (compare Figure 8a vs. h). The *Olig1^Cre/±^;Ascl1*‐CKO mice were able to survive postnatally and continued to exhibit a decrease of OLIG2+ cells in the WM at P14 (Figure 8l). Quantification of the number of OLIG2+ cells/mm^2^ in the GM revealed that it was similar between control (622 ± 35) and *Ascl1*‐CKO (594 ± 64) at P0 but was slightly decreased in the *Ascl1*‐CKO at P14 (797 ± 43 *Ascl1*‐CKO vs. 883 ± 42 control), though this decreased was not significant (blue vs. green bars, Figure 8o). In the WM, the number of OLIG2+ cells/mm^2^ was decreased by about 40% in the *Ascl1*‐CKO compared with control at both P0 and P14 (blue vs. green bars, Figure 8o). This decreased in OLIG2+ cells in the WM but not GM was similar to our previous study in which *Ascl1* was conditionally knocked out in OLPs at E14.5 using *Ascl1^CreERT2^* (Nakatani et al., 2013; Vue et al., 2014).

To determine if the differentiation of NG2‐glia was affected by the complete loss of ASCL1, we next performed immunofluorescence for OL‐differentiation markers for spinal cords at P14. We found that both CC1 and MBP are still expressed in the GM and WM of *Ascl1*‐CKO (Figure 8m,n) as in the control (Figure 8f,g), albeit in fewer cells in the WM due to the decrease in OLIG2+ cells. Collectively, these findings further support a role for ASCL1 to ensure the proper generation and proliferation of NG2‐glia but not their differentiation into myelinating OLs.

## 4 DISCUSSION

The generation, development, and differentiation of NG2‐glia in the CNS is a complex process across both space and time, and requires the precise regulation and function of multiple genes, particularly those encoding transcription factors (*Olig1/2, Nkx2.2, Ascl1, Sox10, Prox1, Smarca4/Brg1*, *Myrf, and Sox17*; Hornig et al., 2013; Kato et al., 2015; Li, Lu, Smith, & Richardson, 2007; Lu et al., 2002; Stolt et al., 2004; Sugimori et al., 2007; Sugimori et al., 2008; Yu et al., 2013; Zhou, Choi, & Anderson, 2001; Zhou & Anderson, 2002; Zhu et al., 2014). Gain‐of‐function and loss‐of‐function studies in the last couple of decades have been informative in revealing the general mechanisms of how OLPs are specified by Sonic hedgehog (SHH) and the various transcription factors to give rise to NG2‐glia and eventually myelinating OLs (Rowitch & Kriegstein, 2010). However, the highly proliferative and migratory behavior of NG2‐glia have made it difficult to fully discern the extent of their heterogeneity and function in different regions of the CNS. At present, it remains unclear how much the various transcription factors contribute to the heterogeneity and development of NG2‐glia in the GM and WM, or if the heterogeneity of NG2‐glia is completely determined by the surrounding landscape.

### 4.1 Differential level of ASCL1 and lineage restriction of OLP/NG2‐glia in the GM and WM of the spinal cord

**Figure 9:**
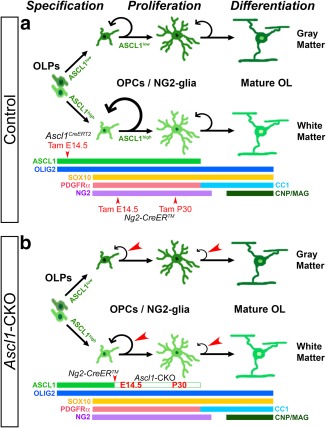
Summary of findings. (a) Schematics of gene expression and developmental dynamics of NG2‐glia in the GM and WM of the spinal cord. *Ascl1^CreERT2^* and *Ng2‐CreER^TM^* labeling mark temporally distinct lineages of NG2‐glia. (b) *Ng2‐CreER^TM^* mediated *Ascl1*‐CKO during embryonic development at E14.5 or in the adult spinal cord at P30 resulted in a reduction of NG2‐glia proliferation (red arrowheads) but not their differentiation into mature oligodendrocytes [Color figure can be viewed at http://wileyonlinelibrary.com]

We observed that one of the first fundamental differences between GM and WM NG2‐glia in the spinal cord is the level of ASCL1 expression. Indeed, by quantifying the immunofluorescence of ASCL1 specifically in OLIG2+;PDGFR+ NG2‐glia, we found that it is twice as high in WM NG2‐glia than in GM NG2‐glia (Figure 1g,r). This difference was apparent from embryonic development up until a month of age, was specific to ASCL1 but not OLIG2 (not shown), and is suggestive that the high and low levels of ASCL1 is a direct reflection of the diversification and heterogeneity of NG2‐lineage cells in the WM and GM, respectively (Figure 9a). Several lines of evidence support this interpretation. First, in loss of function studies, *Ascl1*‐mutant mice, though neonatal lethal, exhibit a dramatic decrease in the number of OLIG2+ cells that are generated in the WM (Sugimori et al., 2007; Sugimori et al., 2008). Similarly, conditional knock‐out of *Ascl1* in glial progenitors in the embryonic spinal cord or neonatal brain resulted in a significant and persistent reduction in the number of OL‐lineage (OLIG2+ and SOX10+) cells that are generated in the WM (Figure 8; Nakatani et al., 2013; Vue et al., 2014). In some of these instances, generation of OLIG2+ cells in GM was not as adversely affected, which might be expected since WM NG2‐glia express a higher level of ASCL1 and therefore may be more dependent on ASCL1 for generation than GM NG2‐glia.

The second line of evidence comes from studies which demonstrate that there are intrinsic differences between NG2‐glia in the GM and WM in terms of gene expression, electrophysiological properties, proliferation and differentiation rates, and response to PDGF or the surrounding environment (Chittajallu et al., 2004; Dimou et al., 2008; Hill et al., 2013; Kang et al., 2010; Psachoulia et al., 2009; Vigano et al., 2013). Whether these differences are directly determined by or attributable to the differential level of ASCL1 remains to be tested. Nevertheless, they are an indication that GM and WM NG2‐glia are distinct and may be derived from separate OLP populations. In agreement with this, our clonal analysis of the spinal cords of *Ascl1^CreERT2^;R26R^LSL‐Confetti^* double transgenic mice reveal that when individual ASCL1+ OLPs are sparsely labeled at E14.5, they form clones of SOX10+;Confetti+ cells that are restricted to the GM or WM at one month of age (Figure 2). This restriction was noted for small cell clones, which are probably derived from more mature ASCL1+ OLPs or NG2‐glia, as well as clones that are comprised of hundreds of cells and are likely to be derived from OLPs in the ventricular zone (Figures 2d,e and 7a). Particularly noteworthy is that the WM clones on average contain more cells per clone than the GM clones. This could be due in part to the higher expression of ASCL1 in WM NG2‐glia, given that the proliferation of NG2‐glia as measured by their ability to incorporate BrdU is reduced in the absence of *Ascl1* (Figure 5). Interestingly, we also observed that the WM contained more than 70% (21/29) of the clones that were labeled, while the remaining clones (8/29) were found in the GM (Figure 2e). This uneven labeling distribution could be explained by the fact that ASCL1 marks two distinct OLP‐lineages in the spinal cord. For instance, ASCL1 is expressed at a higher level in OLPs, as well as NG2‐glia, that are fated to the WM than those that are fated to the GM (Figure 7a), thereby increasing the probability of labeling WM clusters as seen in *Ascl1^CreERT2^; R26R^LSL‐Confetti^* spinal cords. Alternatively, it is possible that there are simply more WM OLP‐lineage cells than GM OLP‐lineage cells, therefore more WM clones are labeled. Regardless, to truly determine if the differential levels of ASCL1 control the specification of OLPs and the diversification and heterogeneity of NG2‐glia in the GM and WM, further experiments are required.

### 4.2 Loss of ASCL1 decreases the proliferation of NG2‐glia in the embryonic and adult spinal cord

Previous studies showed that loss of ASCL1 resulted in reduced OPC generation in the spinal cord at the onset of oligodendrogenesis (Sugimori et al., 2007; Sugimori et al., 2008), and in the first few weeks following conditional deletion of *Ascl1* in OLPs embryonically (Vue et al., 2014). We extend these findings and provide insight into the long‐term effect of *Ascl1* deletion specifically in NG2‐glia during embryonic and adult spinal cord oligodendrogenesis. We observed a significant, persistent reduction in the number of NG2‐glia derived tdTOM+ cells in the GM and WM of the spinal cord following conditional deletion of *Ascl1* at E14.5 or P30 (Figures 4c and 5c). The reduction of BrdU incorporation in the tdTOM+ cells indicates that this decrease in cell number is due to decreased proliferation, although it is possible that an increase in cell death may also occur for NG2‐glia in the absence of ASCL1. This interpretation was supported by long‐term clonal lineage analysis of NG2‐glia clones, which on average contain not only more cells per clone but also more clones overall in the presence rather than the absence of ASCL1 (Figure 7).

Interestingly, in contrast to the observed effect on the number of OLIG2+;tdTOM+ NG2‐glia in the embryonic and adult spinal cord following *Ascl1* deletion, the proportion of these labeled NG2‐glia in the GM or WM that had differentiated into mature CC1+ OLs was not significantly altered at any time point analyzed (Figures 4 and 5). These results are in stark contrast with a prior report showing a drastic reduction in the number of myelin‐expressing OLs in the WM of the spinal cords of *Ascl1*‐mutant mice at P0, a finding that was attributed to impaired or delayed differentiation of *Ascl1*‐mutant NG2‐glia (Sugimori et al., 2007; Sugimori et al., 2008). These contrasting findings suggest that while ASCL1 may be required to ensure proper generation and differentiation of NG2‐glia, the direct long‐term consequences of ASCL1 loss are on proliferation, and potentially survival, but not differentiation of NG2‐glia in both the embryonic and adult spinal cord (Figure 9b). This finding is not surprising considering that key factors such as NKX2.2, OLIG1/2 and SOX10, which have been shown to be crucial for NG2‐glia differentiation, are still present and are not affected by the loss of ASCL1. In contrast, *Olig1^Cre^* conditional knock‐out of *Pdgfrα* resulted in a drastic decrease in the number of OPC/NG2‐glia in the spinal cord (Zhu et al., 2014), a phenotype that is similar but more severe than the *Olig1^Cre^;Ascl1*‐CKO observed in this study (Figure 8). Because PDGFRα expression is unaffected by ASCL1 loss (Figure 8k), this suggests a possible mechanism by which ASCL1 functions in parallel or downstream of PDGFRα to regulate NG2‐glia proliferation.

### 4.3 Potential mechanism and targets of ASCL1 function in NG2‐glia

A fundamental question that needs to be addressed is how ASCL1 differentially regulates NG2‐glia proliferation in the GM and WM. Our finding that higher level of ASCL1 is correlated with a higher proliferation rate of NG2‐glia in the WM over the GM, and in embryonic and neonatal stages over adult stages implies that the level of ASCL1 is critical for controlling the differential proliferation of NG2 glia. This implication of different levels of ASCL1 controlling its function within progenitor cells has been previously described. For instance, time‐lapse imaging of brain slices or neural progenitors in culture revealed that the level of ASCL1, along with OLIG2 and HES1, when fused with reporter proteins oscillates within the cells. Moreover, sustained high levels of ASCL1 promotes cell fate specification and differentiation, whereas lower levels of ASCL1 oscillation promotes cell division (Imayoshi et al., 2013). In agreement with this, ASCL1 expression in neural progenitors in the ventricular zone, whether during neurogenesis or gliogenesis (Figure 1a–e), is relatively high, and may allow ASCL1 to function to ensure proper specification and differentiation of interneurons and OPCs/NG2‐glia (Helms et al., 2005; Parras et al., 2004; Parras et al., 2007; Sugimori et al., 2007). In contrast, the overall level of ASCL1 in NG2‐glia in the GM and WM, though can be readily detected in neonatal, and to some extent, adult stages (Figure 1f–j,r), is much lower and may only be at levels sufficient to promote cell division but not differentiation. This would explain how a higher level of ASCL1 in WM NG2‐glia would promote these cells to proliferate at a faster rate than GM NG2‐glia (Figure 2), and the lack of ASCL1's role in NG2‐glia differentiation.

ASCL1, like other bHLH proteins, functions by forming heterodimers with ubiquitously expressed E‐protein (E47) to bind to DNA to regulate gene expression (Castro et al., 2006). Currently, it is unknown whether ASCL1 dimerizes with E47 or different dimerization partners to differentially regulate the proliferation of NG2‐glia in the GM and WM. However, since ASCL1 shows strong preferences in binding to canonical E‐box (CANNTG) motifs in the genome of highly proliferative neural progenitors and cancer cells (Castro et al., 2006; Borromeo et al., 2014; Borromeo et al., 2016), it is likely that E47 may also be a dimerization partner in NG2‐glia. Recently, a critical role for ASCL1 in regulating neural progenitor cell proliferation has been reported. In particular, Castro et al. (2011) showed that although ASCL1 directly regulates genes critical for the specification and terminal differentiation of neural progenitors in the embryonic ventral telencephalon, a number of direct transcriptional targets also include positive cell cycle genes and oncogenic transcription factors such as *E2f1, FoxM1, Cdca7, Tead1/2*, and *Taz*. More recently, Garcez et al. (2015) also demonstrated that ASCL1 directly regulates *Cenpj/CPAP*, a gene that is critical for centrosome formation and normal mitosis in neural progenitors. Given ASCL1's specific role in regulating NG2‐glia proliferation, it is possible that some of these genes may also be directly or differentially regulated by ASCL1 in NG2‐glia in the GM and WM of the spinal cord, and in other regions of the CNS.

## 5 CONCLUSIONS

Taken together, our lineage tracing analysis at both the population and clonal level provides new insight into the developmental dynamics of NG2‐glia, enabling comparison of long‐term NG2‐glia development in the embryonic versus adult spinal cord and in the GM versus WM. More importantly, our findings suggest that ASCL1 is a critical regulator of the proliferative capacity of GM and WM NG2‐glia in both the embryonic and adult spinal cord. These results highlight a role for ASCL1 in balancing OPC/NG2‐glia proliferation and maintenance with myelin remodeling in the spinal cord throughout adult life. Indeed, it is possible that ASCL1's expression may underlie the local induction of NG2‐glia proliferation and differentiation by neuronal activity that gives rises to OLs capable of re‐myelinating projection neurons, a step that was recently shown to be critical for the learning of complex motors tasks in mice (Gibson et al., 2014; McKenzie et al., 2014; Xiao et al., 2016). Lastly, prior studies have suggested that NG2‐glia represent a potential cell of origin for some gliomas, such as glioblastoma multiforme, an aggressive, treatment‐resistant brain tumor characterized by high ASCL1 expression (Lei et al., 2011; Liu et al., 2011). An understanding of ASCL1's role in regulating the proliferation of NG2‐glia may therefore provide new insights into the formation and progression of these cancers.

## CONFLICT OF INTERESTS

The authors declare no competing financial interests.

## AUTHOR CONTRIBUTIONS

T. Y. V and D. K. designed and performed all experiments and data analysis with assistance from E. H. and M. E. ‐F., and prepared the manuscript with J. E. J. All authors provided scientific insights and edited the manuscript.
